# Function Trumps Form in Two Sugar Symporters, *LacY* and *vSGLT* †

**DOI:** 10.3390/ijms22073572

**Published:** 2021-03-30

**Authors:** Jeff Abramson, Ernest M. Wright

**Affiliations:** Physiology Department, David Geffen School of Medicine at UCLA, Los Angeles, CA 90025, USA; ewright@mednet.ucla.edu

**Keywords:** symporters, lactose permease SGLTs, structure, function

## Abstract

Active transport of sugars into bacteria occurs through symporters driven by ion gradients. **LacY** is the most well-studied proton sugar symporter, whereas *vSGLT* is the most characterized sodium sugar symporter. These are members of the major facilitator (MFS) and the amino acid-Polyamine organocation (APS) transporter superfamilies. While there is no structural homology between these transporters, they operate by a similar mechanism. They are nano-machines driven by their respective ion electrochemical potential gradients across the membrane. **LacY** has 12 transmembrane helices (TMs) organized in two 6-TM bundles, each containing two 3-helix TM repeats. *vSGLT* has a core structure of 10 TM helices organized in two inverted repeats (TM 1–5 and TM 6–10). In each case, a single sugar is bound in a central cavity and sugar selectivity is determined by hydrogen- and hydrophobic- bonding with side chains in the binding site. In vSGLT, the sodium-binding site is formed through coordination with carbonyl- and hydroxyl-oxygens from neighboring side chains, whereas in **LacY** the proton (H_3_O^+^) site is thought to be a single glutamate residue (Glu325). The remaining challenge for both transporters is to determine how ion electrochemical potential gradients drive uphill sugar transport.

## 1. Introduction

Cotransport was first proposed for animal cells by Bob Crane and shortly thereafter extended to bacteria by Peter Mitchel where the process was renamed symport [1,2]. It is now well established, that the energy for active transport of sugars, and other substrates, is provided by ion electrochemical potential gradients across the cell membrane, which are primarily protons in bacteria and sodium in animal cells. Two of the best-studied sugar transporters in bacteria have been the proton/lactose symporter (*LacY*) by Kaback lab and sodium/glucose (*vSGLT*s) symporter by the Wright and Abramson labs [3,4,5,6,7,8]. *LacY* and *vSGLT* are members of two distinct super gene families referred to as the MFS (Major Facilitator Superfamily) and APC (Amino acid Polyamine-organoCation) superfamilies (2.A.1.5.1 and 2.A.21 in the transporter classification database, http://www.tcdb.org, accessed on 26 February 2021). The *E. coli* *LacY* and *Vibrio parahaemolyticus* SGLT sugar transporters were amongst the first to be cloned, sequenced, functionally characterized, and crystalized [3,4,5,6,7,8,9,10,11,12,13,14,15,16]. Although there is no amino acid sequence or structural homology, they exhibit common transport properties. This review compares the form and function of *LacY* and *vSGLT* to gain insight into their transport mechanisms.

## 2. Lactose and Glucose Transport

**Figure 1:**
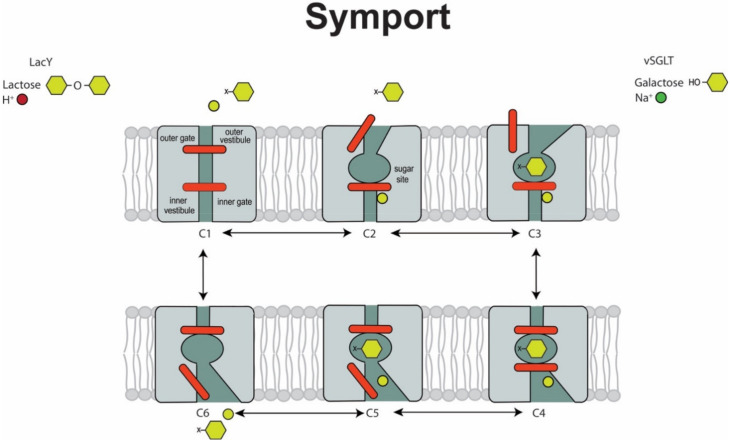
A model for H^+^/lactose and Na^+^/glucose transport by *LacY* and *vSGLT*. The transporters are shown as double-gated integral membrane proteins with a minimum of 6 states that undergo a series of ordered reversible reactions that drive inward sugar transport. The first step is the binding of external cation (H_3_O^+^ O or Na^+^ O) that results in the opening of the external gate (C1 to C2) to allow sugar to bind (C2 to C3). This is followed by closure of the external gate to occlude the bound sugar from both sides of the membrane (C3 to C4). The subsequent opening of the internal gate permits the release of sugar and cation into the cytoplasm (C5 to C6) and the return of the protein into the initial conformational state (C6 to C1). The net result is the obligatory coupling of the inward transport of one cation and one sugar molecule across the membrane into the cell. The rate and direction of sugar transport depends on the concentrations of ions (proton or sodium) on each side of the membrane, and the voltage across the membrane.

The transport characteristics of the H^+^/lactose (*LacY*) and Na^+^/glucose (*vSGLT*) symporters can be summarized in terms of the mechanical model shown in Figure 1. The transporters, depicted as monomers in the plasma membrane transition through at least 6-conformational states as they transport their cargo. Protons are unique in their ability to drive lactose transport by *LacY*, while sodium is the preferred cation for *vSGLT* with an apparent sodium *K*_m_ in the range of 5–10 mM.

Uphill transport occurs by obligatory coupling of sugar and cation transport by an alternative access mechanism where external cation binds first to open the “external gate” (C1 to C2), permitting external sugar to bind to the transporter in the outward open conformation (C2 to C3). The outer gate then closes to trap sugar in an occluded state (C4) before the “inner gate” opens to allow the release of sugar and cation into the cytoplasm (C5 to C6). The transporter finally reverts to the starting outward conformation to complete the cycle (C6 to C1). The net result is that 1 cation and 1 sugar are transported across the membrane in each transport cycle. The rate limiting step for inward symport is the release of H^+^ and Na^+^ to the cytoplasm (C4 to C5) [1,17]. Each complete cycle takes about 0.05 seconds to complete.

These transporters are totally reversible, where the direction and rate of transport is simply determined by the sum of the activity of ions (the electrochemical potential) and sugar (the chemical potential) on each side the membrane. Both transporters are electrogenic, i.e., transport generates a proton or a sodium current. With equal cation and sugar concentrations on each side of the membrane, the rate and direction of sugar transport simply depends on the polarity and magnitude of the voltage across the membrane.

## 3. Transported Sugars

Lactose is the natural substrate for *LacY* with a *K*_m_ of 1 mM. Other lactose analogs, e.g., β-D-galactopyranosyl-1-thio-β-D-galactopyranoside (TDG) and 4-nitrophenyl-α-D-galactopyranoside (α-NPG), are also slowly transported but with higher affinity [3]. In addition, *LacY* is capable of transporting galactose with a low affinity but is unable to transport glucose or glucosides. On the other hand, *vSGLT* transports both glucose and galactose (*K*_m_ 0.2 mM), while disaccharides are not transported and glucopyranosides are either not transported or behave as inhibitors, e.g., phlorizin *K*_i_ 1 mM [15]. The inhibition of human SGLTs by phlorizin has been exploited by the pharmaceutical industry to develop high affinity phlorizin analogs for the treatment of Type 2 Diabetes Mellitus [18,19]. Many other uniporters, symporters and exchangers in the MFS and APC gene families share a similar alternating access mechanism with variations in the cation/substrate coupling (0 to 3) and may be electrogenic or not. For example, the human glucose transporter GLUT1 is neither ion coupled nor electrogenic [20].

## 4. Biochemistry

The MFS and APC genes are expressed in all life forms. The *LacY* gene was the first member of the MFS family to be cloned and sequenced [3]. The predicted 46.5 kDa protein contains 12 transmembrane α-helices [3]. Similarly, the *vSGLT* gene was the bacterial member of the APC family to be cloned and sequenced [4,10]. The predicted 58.9 kDa protein contains 14 transmembrane α-helices [4,11]. The APC family members range anywhere from 11–15 transmembrane α-helices [11]. Prior to solving the atomic structures extensive biochemical work confirmed the predicted mass and α-helical content of *LacY* and vSGLT, e.g., by electron-spray ionization mass (ESI-MS) and circular dichroism (CD) spectroscopy [4,16,21]. These transporters are fully functional as monomers, but they also share a remarkable property in that when the N- and C- halves are expressed independently (in the same cell) they form fully functional proteins. This demonstrates that the split proteins can assemble into fully functional transporters in the plasma membrane [3,12].

## 5. Overall Structure

The structure of *LacY* was the first symporter to be determined [9]. Subsequently structures of other conformations, outward-open, inward-open, and partially occluded sugar bound, have been resolved [22,23,24,25,26,27]. Crystal structures of *vSGLT* have been solved in the inward open and the inward open sugar occluded conformations [5,6]. Homology models are also available for the outward facing SGLT conformations based on the outward facing structure of the Proteus mirablis salic-acid transporter SiaT [28]: *vSGLT* and SiaT share 24% sequence identity and 40% similarity [8,29]. The fidelity of outward facing homology models were tested by functional assays on mutated essential residues [29].

**Figure 2:**
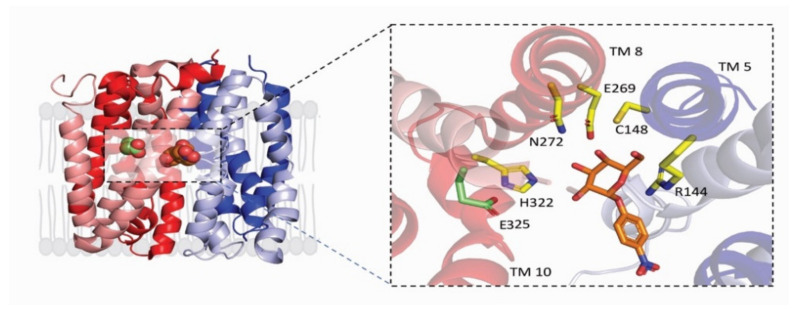
The core domain of the inward-occluded conformation of *LacY* (PDB: 4ZYR). Side view of *LacY* in the plasma membrane highlighting the α-NPG binding site. In this orientation, the cytoplasmic side of the protein is up. The characteristic 6TM repeat is shown in blue (TM1-TM6) and red (TM7-TM12) with the putative protonation site E325 (green) and substrate NPG (orange) spheres. Inset, shows the location of the protonation site E325 (green) in relation to NPG (orange) with their corresponding coordinating residues. The galactosyl subunit of α-NPG is coordinated by H-bonds to side chains on TMs 5, 8 and 10, and hydrophobic stacking to Tyr256 on TM7. Note that two “kinked” (unwound) helices, TM7 and TM10, help accommodate the sugar in the binding site. Atoms are displayed in ball-and-stick form with oxygen colored (red) and nitrogen colored (blue).

**Figure 3:**
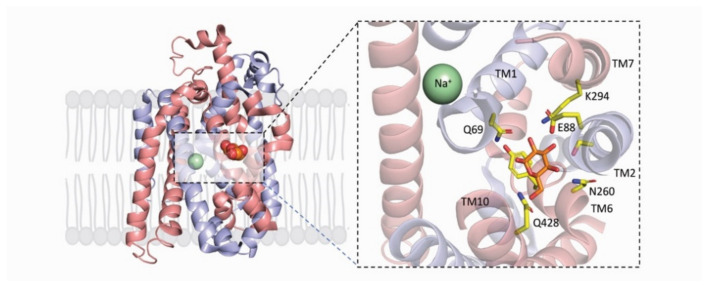
The core domain of the inward-occluded conformation of *vSGLT* (PDB: 3DH4). The characteristic 5TM inverted repeat is shown in blue (TM1–TM5) and red (TM6–TM10) with the driving ion Na^+^ (green) and substrate galactose (orange) spheres. Inset, shows the location of Na^+^ (green) in relation to galactose (orange) with their corresponding coordinating residues. The sugar is accommodated in the middle of the membrane by a space created in part by “kinked” (unwound) helices TM1 and TM6. As with *LacY*, the sugar is coordinated by H-bonds to polar side chains on TM1, TM2, TM6, TM7 and TM10) and hydrophobic stacking of the pyranose ring to Trp263. The presumptive sodium binding site is located between TM1 and TM8 some 10 Å distant from the sugar binding site. Atoms are displayed in ball-and-stick form with oxygen colored (red) and nitrogen colored (blue).

The X-ray structures of *LacY* and *vSGLT* share several features in common in that they are polytopic integral membrane proteins with 12–14 irregular transmembrane helices with distorted bends and kinks (Figure 2). In *LacY*, the 12 TM helices are organized into two six-helix bundles with a long cytoplasmic loop between TM6 and TM7. Within each six-helix bundle, there are two three-helix inverted repeats. There is an obvious aqueous channel stretching from the cytoplasm to the empty sugar binding site (see below). In subsequent structures, the sugar α-NPG was observed in this central cavity (Figure 2). In the initial structure of *vSGLT*, it was found to have 14 irregular trans-membrane helices with galactose bound in a central occluded cavity (Figure 3). Just below the bound sugar, there is an aqueous channel that leads to the cytoplasm (see below). *vSGLT* contains two five transmembrane helices in an inverted repeat, TM1–TM5 and TM6–TM10, that is conserved within the APC superfamily. In order to ease comparison of the inverted repeat, between members of the APC family, the *vSGLT* helices were renumbered from TM-1 to TM13 [30]. Although there is no amino acid homology between the five-TM repeats in *vSGLT*, or any other APC proteins, there is a close structural homology between the repeats (RMSD 4 Å). The inverted repeat is related by a 153° rotation parallel to the membrane plane. Two helices from each repeat, TM1, TM2 and TM6, TM7, surround the bound sugar (the sugar or bundle domain) and this is surrounded by two helices from each repeat, TM3, TM4 and TM8, TM9 (the scaffold domain). Two long helices from each domain, TM5 and TM10, are referred to as the gating domain, connects the sugar and scaffold domains. There is high structural homology between this pair of helices in each inverted repeat (RMSD 0.9 Å). Finally, each protein contains several short extramembrane helices (Figure 3) that have been implicated in the regulation of transport in other MFS and APC proteins.

## 6. Sugar Binding Sites

In both *LacY* and *vSGLT*, a single sugar binding site was identified midway across the protein (Figure 2), whose architecture remains fixed and independent of sugar occupancy and protein conformation [3,5,6,8,29]. In each case, the galactosyl polar groups are hydrogen-bonded to polar side chains on residues lining the binding site, and the pyranose ring stacks with the ring structures of a neighboring tryptophan (*LacY*) or tyrosine (*vSGLT*) residues. The -OH groups of galactose in the *LacY* structure are hydrogen bonded to Tyr256 (TM7) Glu269 (TM8), Asn272 (TM8), and His322 (TM10); and in *vSGLT* Q69, (TM1) E88 (TM2), K294 (TM7) N260 (TM6), and Q428 (TM10). Collectively, TM1, TM2, TM6 and TM7 in *vSGLT* is referred to as the “sugar bundle”, while TM3, TM4, TM8 and TM9 are referred to as the scaffold domain.

## 7. Ion Binding

While there is no structural evidence for the location of the H^+^ binding site in *LacY*, there is substantial biochemical evidence. E325 on TM10 (Figure 2) is required to be protonated for lactose symport or exchange and is likely the primary functional binding site [3]. The putative Na^+^ binding site for *vSGLT* is located 10 Å away from the sugars binding site (Figure 3), and Na^+^ is coordinated by 2 hydroxyl side chains, S354 and S355 on TM8 and the carbonyl oxygens of A62 and I65 on TM1 and A361 on TM8. This site, referred to as Na2, is conserved in other members of the APC superfamily. While it is difficult to definitively resolve Na^+^ by- X-ray crystallography, there is substantial biochemical and biophysical data supporting its location [5,6,31].

## 8. Sugar Access to Its Binding Site

**Figure 4:**
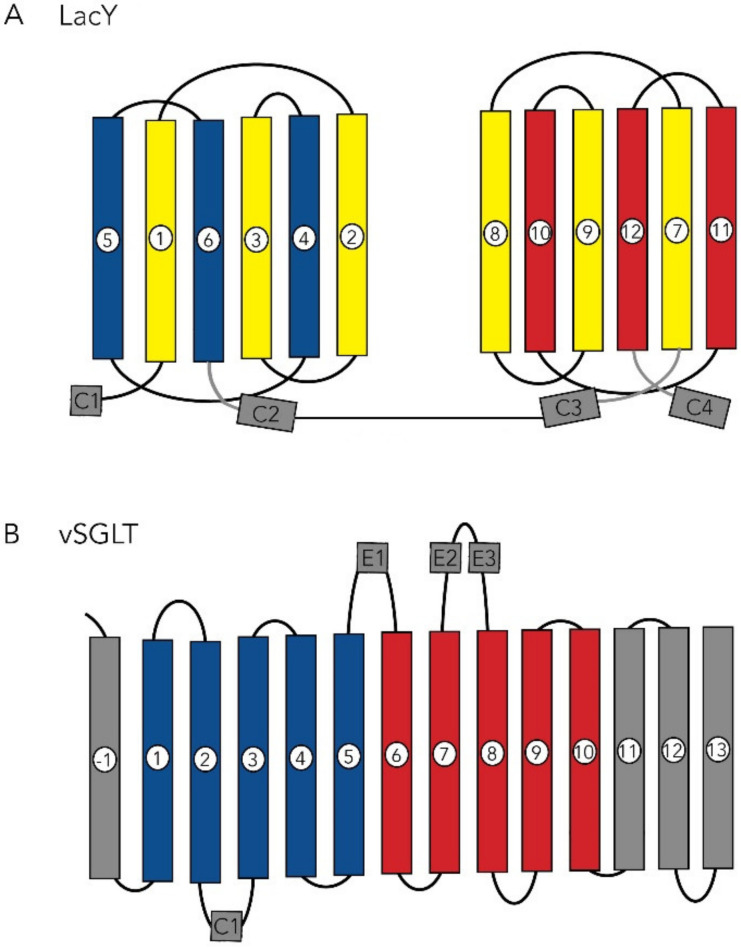
Topology of *LacY* and *vSGLT*. *LacY* shows N-terminal TM 1–6 and C-terminal TM 7–12 domains, and each domain contains a 3TM inverted repeat, a common feature of MFS transporters. The 4 short cytoplasmic helices, C1–C4, are also conserved in members of the MFS family, but there is no evidence that they regulate transport activity in *LacY*. *vSGLT* has 14 TM helices, TM-1 to TM13 and four short extramembrane helices, C1, E1, E2 and E3. In common with other APC transporters, *vSGLT* contains a core of 10 transmembrane helices, TM1–TM10, organized in a 5 TM inverted repeat, TM1–TM5 and TM6–TM10. The numbering of the TM helices is simply to facilitate comparison with other APC transporters (Abramson & Wright 2009).

**Figure 5:**
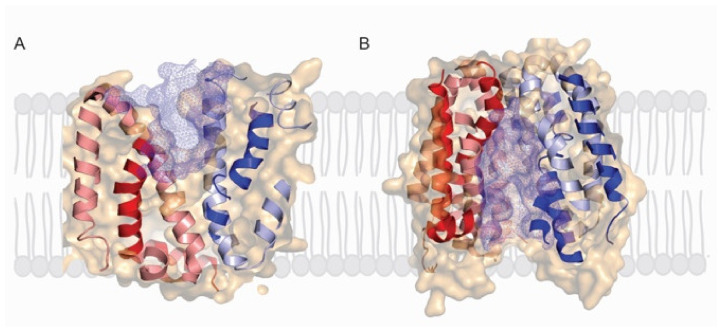
Alternating access of *LacY*. (**A**) Slice through surface of the partial outward structure of *LacY* (PDB: 5GXB) viewed from the membrane plane showing the extracellular cavity (blue mesh). (**B**) Slice through surface of the inward-facing structure of *LacY* (PDB: 1PV6) in the membrane plane showing the intracellular cavity (blue mesh). The characteristic 6TM repeat is shown in blue (TM1–TM6) and red (TM7–TM12) and the surface is shown in beige.

An obvious question regarding the location of the central sugar-binding site is how do sugars enter and leave on each side of the membrane? The answer is that there is coordinated opening and closing of aqueous channels at the external and internal faces of the proteins as they undergo the transport cycle (Figure 1), which is evident from structures in different conformations (Figure 4 and Figure 5). On the extracellular surface, an aqueous channel leads to the sugar binding site in the outward facing conformation while the intracellular channel is closed. Although it has been a challenge to obtain the structures of *LacY* and *vSGLT* in the outward-facing confirmation, a *LacY* double tryptophan mutants (G46W/G262W) [22,23,24,25,26,27] and homology models [8,29] (see Figure 4A and Figure 5A) point to these transitions. The *LacY* external channel opening to the sugar binding site is narrow (4 Å) diameter and lined by the external ends of TM1, TM4, TM7 and TM10. The crystal structure shows a constriction at F27, yet there is rapid binding of oversized external galactosides to the *LacY* sugar binding site, which stabilize an outward-open conformation [32]. This emphasizes the conformational flexibility of the extracellular aqueous channel, and the limitations on the interpretation of static crystal structures.

**Figure 6:**
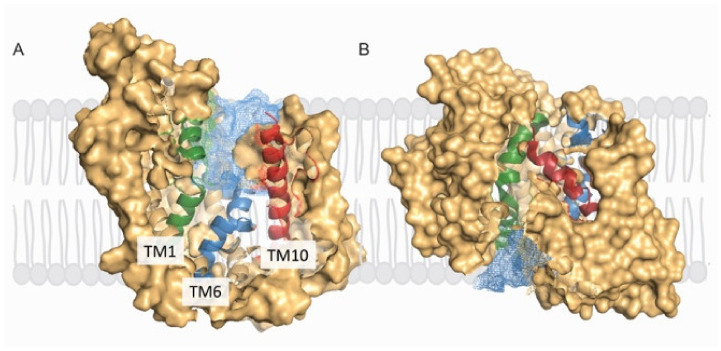
Alternating access of *vSGLT*. (**A**) Slice through surface of the outward-facing model viewed from the membrane plane showing the extracellular cavity (blue mesh) [8,29]. (**B**) Slice through surface of the inward-facing structure of *vSGLT* (PDB: 3DH4) in the membrane plane showing the intracellular cavity (blue mesh). Helices showing large structural rearrangement are colored green (TM1), blue (TM6) and red (TM10) with the surface is shown in beige.

The external channel of *vSGLT* is lined by TM1, TM2, TM6 and TM10, leads to the sugar binding site (Figure 6). It is large enough to accommodate the diffusion of galactose and large galactoside inhibitors to the sugar-binding site when it is open in the presence of external sodium [15]. The architecture of the human SGLT isoform has been extensively probed using functional assays, molecular modelling, labelling of glucose binding site cysteine mutants with TAMRA-MTS, and the binding of potent glucoside inhibitors [29,33]. The glucose moiety of the inhibitors binds to the glucose-binding site, and the aglycone occupies the outer hydrophobic vestibule some 8 Å from the substrate-binding site. After sugar binding, the external channel closes by an inward tilt of the external end on TM10 towards TM1e and TM2e, and the collapse of extracellular loops E2 and E3 into the channel (Figure 3). The back flux of bound galactose into the extracellular solution is also impeded by a hydrophobic gate formed by M73 (TM1), Y87 (TM2) and F424 TM10) [5]. This ‘seal’ is not perfect as molecular dynamic studies indicate that it is permeable to water and galactose, but not sodium. Galactose back flux to the extracellular side, through this gate, occurs at a rate about 10% of that to cytoplasmic surface [7]. The extracellular galactose escape is due side chain motions of M73, Y87 and F424 rather than global conformational changes.

The cytoplasmic aqueous channel leading from the lactose binding site to the cytoplasm is evident from multiple crystal structures (Figure 4B). This 25 Å deep by 10 Å wide channel is lined by the internal ends of TM2, TM5, TM8, and TM11. In biochemical studies, with *LacY* locked in the inward facing conformation, substrates on the external surface do not access the lactose binding site, whereas substrates on the cytoplasmic surface have unrestricted access to down this channel [32,34].

The cytoplasmic channel of *vSGLT* is formed by the internal ends of TM1, TM2, TM3, and TM7. After Na^+^ and galactose release the volume of the channel increases by ~1500 A^3^ due in part to a 6° rigid rotation of the sugar and scaffold domains and the outward flexing of the cytoplasmic end of TM1 by ~13° [6]. Extensive molecular dynamic studies of galactose release from the crystal binding site shows that the sugar freely exits to the cytoplasm by diffusion down this channel [6,7,35]. In the human SGLTs, large glucosides, e.g., phlorizin, are not able to enter the sugar binding site from the cytoplasm site as judged by the lack of phlorizin inhibition and binding [29].

## 9. Isomerization between the Outward and Inward Conformations

While there is overwhelming biochemical and structural evidence for alternating access model for lactose and glucose symport (Figure 1), we require a further structural analysis of the conformational changes between the outward facing ligand bound and inward facing ligand-free states (Figure 4 and Figure 5). The objective is to arrive and test mechanical symport models, as exemplified by those for the F_1_/F_o_ ATP synthase [36]. For symporters and antiporters there is active consideration of rocking bundle, and elevator models. For *LacY* the rocking bundle model involved a rigid concentric rotation of the N- and C-terminal domains by 16° and local changes in the two kinked helices TM7 and TM10 (Figure 4). Whereas for APC transporter the rocking bundle model involves a 29° rotation of the scaffold (TM3, TM5, TM8, and TM9) domain relative to the hash (TM1 TM2, TM6 and TM7) domain, and local changes in TM5 and TM10) (Figure 5). These transitions are in generally consistent with biochemical and biophysical data [3,8], but there are obviously oversimplifications with current models.

## 10. Conclusions

This survey of the structure and function of *LacY* and *vSGLT*—two sugar symporters in different gene families with different structural folds—has highlighted their very close functional similarities. Both transporters share kinetic properties: ligand binding is ordered, cation first; transport of ion and sugar is strictly coupled with prohibited leakage of cation or sugar; the process is electrogenic; and that the rate and direction of transport is simply determined by the sum of the driving forces (chemical and electrochemical potentials). Uphill (active) transport of each ligand can simply be driven by gradients of the partner ligand, or by voltage in the absence of ligand concentration gradients, i.e., they behave as perfect thermodynamic machines. Both transporters are monomers with a central sugar binding site with aqueous channels leading to their sugar binding sites from the external and cytoplasmic solutions. It is the reciprocal opening and closing of these channels that are key structural basis for the alternating access transport model (Figure 1). Binding of external cations, H^+^ or Na^+^, cause the external vestibule to open and permit sugar binding that in turn leads successively to the closure of external channel and opening of the cytoplasmic channel. While the turnover of the transport cycles is slow, ~20 s^−1^, cation binding is fast, approaching diffusion limitation, and the activation energy for ligand binding is low (<5 kcal/mole). The isomerization of the loaded and unloaded carriers is relatively slow, 50 s^−1^, but the activation energy is between 25 and 30 kcal/mole. What is not known at present is how ligand binding produces these ordered conformations changes.

Clues have emerged about mechanisms from the subtle changes in atomic structure of the protein in different conformations, and more will certainly come from structures in all the conformational states (Figure 1). Thus far, in the case of LeuT, the foremost member of the APC superfamily, atomic structures have been in reported for five intermediate state [37] but little information is available about the probability of these states occurring in the natural transport cycle. Further progress will require functional data on each step in the transport cycle. This may be achieved by CryoEM of proteoliposomes where the symporters are held in intermediate states by a judicious combination of external and internal ligand and inhibitor concentrations and a controlled membrane potential. Dynamic information has already been obtained for partial steps in the transport cycle using stop flow [38], and perturbation methods such as voltage-jumps [1,31,33]. Finally, we conclude that much has to be gained by functional studies on symporters, antiporters and uniporters with differing structural motifs. *LacY* and *vSGLT* share common functional properties but have different structural folds, whereas *LacY* and GLUTs share a common structural fold while the former is a symporter and the latter are uniporters.
